# University students’ access to mental health services: A qualitative study of the experiences of health service professionals through the lens of candidacy in England

**DOI:** 10.1177/13558196241235877

**Published:** 2024-02-27

**Authors:** Tom G Osborn, Rosa Town, Majeed Bawendi, Emily Stapley, Rob Saunders, Peter Fonagy

**Affiliations:** 1 ARC Research Assistant, Research Department of Clinical, Educational and Health Psychology, 4919 University College London, London, UK; 2 Digital Community Manager, The Tavistock and Portman NHS Foundation Trust, London, UK; 3 MBBS Student, UCL Medical School, 4919 University College London, London, UK; 4 Senior Research Fellow, Evidence Based Practice Unit, 4919 University College London, London, UK; 5 Associate Professor, CORE Data Lab, Research Department of Clinical, Educational and Health Psychology, 4919 University College London, London, UK; 6 Professor, Division of Psychology and Language Sciences, 4919 University College London, London, UK

**Keywords:** candidacy, access, student mental health

## Abstract

**Objectives:**

In order to develop a better understanding of students’ access to mental health services, we explored the experiences of health care professionals interacting with university students with mental health problems.

**Methods:**

We interviewed 23 professionals working across university advice and counselling services, NHS general practice, crisis, and psychological services in North and East London between June 2022 and January 2023. Our approach drew on reflexive thematic analysis and the principles of abductive analysis. The notion of candidacy – that is, how different needs are deemed deserving of health service attention – was particularly helpful to our understanding of the ongoing phenomenon of interest in the data.

**Results:**

Each student’s access to mental health support was highly contingent on the student’s dynamic social context and the pressures and organisation of the local health system. Professionals described how different students viewed different needs as deserving of health service attention. Which students reached the professional’s service depended on the resources and relationships a student could draw upon, and the service’s relative permeability. Once there, what action professionals took was strongly influenced by the professional’s service expertise, resource constraints, the relationships the professional’s service had with other organisations, the students’ wishes, and whether students regarded treatment offers as acceptable.

**Conclusions:**

Candidacy offers a useful lens to view university students’ access to mental health support. Access appears to be an increasingly intricate task for students, given the fragmented service landscape, surging demand for mental health care and challenges of emerging adulthood. Our findings suggest that policy goals to increase use of mental health services are unlikely to improve outcomes for students without policy makers and health systems giving holistic consideration of inter-service relationships and available resources.

## Introduction

There have been increasing concerns about the mental health of university students amid a rapidly changing international higher education context. University remains a transition point to adulthood, a period where mental health problems first emerge for many students.^1,2^ Within the current context of higher education, developmental tasks of emerging adulthood may be increasingly challenging for some students.^1^ Many more people attend university with diverse needs, for whom it is increasingly economically costly and academically competitive.^3^ For students who experience mental health problems, mental health services are an important resource for intervention. In the UK, organisations highlight increasing demand for these services and students report access difficulties.^4,5^

In most high-income countries, a range of mental health services exist, but current evidence suggests a large and unequal treatment gap. Epidemiological evidence shows around 30% of students experience symptoms indicative of a mental disorder.^6^ Compared to the general student population, prevalence appears to be increasing among specific groups, such as women, LGBTQ+, and some minoritized ethnic groups.^3,7^ This may be related to differential exposures to risk factors and behaviours during development, such as economic and psychosocial stressors, and discrimination.^8^

Despite increasing prevalence, few students with mental health problems access mental health services. International estimates suggest only around 17% of students with a likely mental disorder have used mental health services within the previous 12 months.^6^ However, this varies considerably across different services, settings, and countries.^9^ Findings also highlight unequal access by student characteristics. For example, women and LGBTQ + groups being more likely to access services, while men, some international students, and minoritized ethnic students may be less likely to access services in some settings.^6,10,11^

Variation in service use may not simply be related to barriers to supply and demand. Studies of university service staff describe how presentations are increasingly severe and complex, sometimes beyond their capacity to support.^12^ Other studies have highlighted problems in structure, organisation, and coordination between health services and universities.^13^ Students describe attempting to navigate a complex and fragmented care system while self-managing their mental health.^14^ Studies highlight the influence of discrimination, confidentiality concerns, stigma, and socio-cultural values, such as self-reliance at universities, in help-seeking.^13,15^ Consequently, many students draw on support from friends and family when in distress.^5,16^

To develop our understanding of how different influences on access to mental health services operate in university students, it is important to acknowledge key gaps in the literature. Firstly, much exploration of this problem has focused, understandably, on students, but research is more limited in terms of those who work in mental health services.^13–15^ Evidence suggests students may access multiple services across a local health system when seeking help, but so far, existing studies have focused on specific services.^9,12^ Secondly, other studies have highlighted limitations of existing theory in understanding access, with much empirical research drawing on theories of help-seeking behaviour.^17^ Other accounts of access emphasise agency, capacity, and social relationships, which literature suggests are important when considering access to mental health services in students.^13,14,18,19^ These findings suggest that theories that take account of health service structures and processes through which students gain access to appropriate mental health support, be that formal or informal, may be relevant.

‘Candidacy’ describes how different needs are deemed deserving of health service attention. This includes how needs are identified, who navigates to and then appears at health services, and how professionals and patients make decisions, influenced by the wider conditions in which services operate.^18,20–22^ Burden of Treatment Theory may also be relevant.^19^ This describes how patient and professional interactions are shaped in modern health systems by the patient’s general capacity to integrate ‘patient work’, which is increasingly delegated to them, in order to ‘self-manage’ their health. Perverse or suboptimal patterns of health service use may arise in situations where capacity to self-manage is overwhelmed.^19^ This may be relevant given many students initially access formal services after ‘self-managing’ through informal interactions.

Therefore, in a theoretically informative way, we aimed to explore experiences of health care professionals interacting with students in mental distress across a range of services these students’ use. Our research questions were:

(1) How do health care professionals understand the mental health problems presented by university students?
(2) What specific actions, if any, do health care professionals take to respond to mental health problems presented by university students?
(3) How are these specific actions perceived by health professionals to be supported in specific work settings?
(4) What are health professionals’ perceived limitations of these specific actions in specific work settings?

## Methods

We conducted a qualitative study using semi-structured interviews sampling health care professionals working in university and National Health Service (NHS) mental health services between June 2022 and January 2023. We drew on abductive analysis for the analytic strategy, reporting in line with the consolidated criteria for reporting qualitative research checklist (see online supplement S1).

### Researchers’ positionality

The lead author (TO) is White British, identifies as a man and has a background in nursing and public health. The primary coding and interpretation was conducted in tandem with a fellow PhD student (RT) who is White American, identifies as a woman and has a background in psychology, and an undergraduate medical student (MB), who is Arab British and identifies as a man. Both TO and RT have previously used university and NHS mental health services. Analysis was enriched through regular meetings with both RS and PF (PF, is an experienced clinician) who identify as men, and an experienced qualitative researcher (ES) who identifies as a woman, all of whom are from White European backgrounds and trained in psychology.

### Recruitment and sampling strategy

The sampling frame for this study was informed by a systematic review,^9^ and a local mapping process to identify services commonly used by students. Research sites centred around two large universities in London. These services were: (1) university advice services; (2) university counselling services; (3) NHS psychological services; (4) crisis services which offer one-off support and assessment, and (5) General Practice. Participants needed to be aged 18 or older, a professional providing care to university students in mental distress, and working with students in London on a part-time basis for a minimum of 6 months.

We first purposively sampled participants through the primary researcher’s existing organisational contacts, starting with university advice and counselling services, and via a pan-London higher education network.^23^ During interviews with professionals from university services, connections to crisis, psychological, and general practice services were repeatedly mentioned. Therefore, we proceeded to recruit from these services. Our second strategy was snowball sampling, where each participant was asked to nominate at least one additional colleague from their service.^24,25^ All potential participants received an email with a participant information sheet and a consent form before choosing to take part. Participants selected a time and date for an interview via a Calendly link. No incentives or compensation were provided.

### Data collection

All participants took part in semi-structured interviews with TO lasting up to 1 hour. Recruitment took place between June 2022 and January 2023, during a time of significant pressures on NHS resources following the COVID-19 pandemic. Therefore, all participants were given the option of being interviewed in person or via Microsoft Teams. All chose the latter option.

A semi-structured interview topic guide of open-ended questions about professional’s role, experiences of managing student mental health difficulties, and perceived drivers of difficulties that students presented to them. All participants were asked to describe in detail three examples: a common student presentation, an uncommon presentation, and a challenging presentation. The examples provided were intended to elicit detailed descriptions of the experiences participants had with students, encompassing the students’ entire help-seeking journey up to the conclusion of the professional-student interaction. Interviews were recorded and transcribed verbatim.

As TO is a health care professional, topic guides were developed with a panel of five members of the National Institute for Health and Social Care Research Applied Research Collaboration (NIHR-ARC) North Thames Virtual Document Review Panel, to ensure questions were sensitive to experiences of those who access services. These individuals all have lived experience of health and social care, living in the region where the study was conducted.

We did not determine a sample size based on data saturation prior to data collection. Rather, throughout sampling, diversity, quality, and richness of accumulated data were monitored through ongoing coding and reflections.^24,25^ We stopped recruitment after 23 participants. This decision was informed by the depth and richness of the data gathered, as well as the diversity across dimensions determined before commencing data collection: service type, professional role, tenure in the current role, and age.^23^

### Data analysis

Data were analysed throughout data collection following principles of abductive analysis. Grounded in pragmatist epistemology, abductive analysis is a qualitative data analysis aimed at theory construction.^24^ Abduction refers an iterative process of developing theoretical insights from unexpected findings then systematically examining how they vary across the dataset.^24^ Throughout data collection, reflexive thematic analysis was used as a first step of analysis to identify central ideas in the dataset.^25^ The methods and results of reflexive thematic analysis are described in full in online supplement S2.

The themes were identified across the interview responses. These themes were presented to 10 study participants in three focus groups in January 2023. Together, we determined the key phenomenon of interest was the work that students, their social relations, and professionals were involved in for students to gain access to appropriate mental health care.

Further, using candidacy as a sensitising lens for the phenomenon, we conducted focused coding. This involved identifying an extract of text, or ‘index case’, which could be used as a comparative benchmark, from the transcripts that clearly affirmed the results’ seven key features: identification, navigation, permeability of services (the ease of service use, based on how well configured services are to the needs of different service users), appearance, adjudications, offers of and resistance to treatment, and local operating conditions.^18,20^ Using the index case as a comparison, we then examined how this phenomenon varied across the dataset and services. We assessed the *fit* of candidacy to the data by actively searching for negative cases, as well as the concept’s *plausibility* by also considering Burden of Treatment.^19,24^ See online supplement S3 for more details on the concept of candidacy and limitations of Burden of Treatment in this analysis.

## Results

### Participants

A total of 35 professionals were approached with 23 agreeing to participate (see Table 1 for a detailed description of the participants). Participants were diverse in terms of age (27 to 75 (mean = 48, SD = 12.9)), tenure in current role (6 months to 17 years (mean = 5.37, SD = 5.3)), service type, and professional role. Of the 13 participants working in university services, five were counsellors, five were mental health advisors with varied professional backgrounds, like social workers and occupational therapists, two were managers, and one a psychiatrist. Of the 10 NHS participants there were three psychologists, three general practitioners, two psychiatrists, and two mental health nurses. Fifty-seven percent (*n* = 13) of our participants were female.

**Table 1.** Participant characteristics.

Participant ID	Professional role	Years of experience in current role	Sector	Service	Size of service population^a^
MHA1	Mental health advisor	3	University	Mental health advice (MHA)	43,900
MHA2	Mental health advisor	1	University	MHA	32,000
MHA3	Mental health advisor	2	University	MHA	32,000
MHA4	Mental health advisor	0.5	University	MHA	4000
MHA5	Mental health advisor	2	University	MHA	2000
CPT1	Psychotherapist	3	University	Counselling	4000
CPT2	Psychotherapist	4	University	Counselling	43,900
CPT3	Psychotherapist	17	University	Counselling	5500
CPT4	Psychotherapist	1	University	Counselling	1000
CPT5	Psychotherapist	8	University	Counselling	1000
MAN1	Manager	3	University	MHA and counselling	2000
MAN2	Manager	2	University	MHA and counselling	32,000
PSYC1	Psychiatrist	16	University	MHA and counselling	43,900
PSYC2	Psychiatrist	12	NHS/University	NHS and MHA	1,600,000^b^
PSY1	Psychologist	1	NHS	Psychological therapy	1,600,000^b^
PSY2	Psychologist	1	NHS	Psychological therapy	1,600,000^b^
PSY3	Psychologist	1	NHS	Psychological therapy	1,600,000^b^
CRIS1	Mental health nurse	4	NHS	Crisis service	2,000,000^b^
CRIS2	Psychiatrist	2	NHS	Emergency department and liaison psychiatry	1,600,000^b^
CRIS3	Mental health nurse	8	NHS	Crisis service and liaison psychiatry	2,000,000^b^
GP1	General practitioner	11	NHS	General practice	7000^b^
GP2	General practitioner	10	NHS	General practice	20,500^b^
GP3	General practitioner	4	NHS	General practice	20,500^b^

^a^Service population estimated based on universities’ published figures of the total student population, or the integrated care system’s published population, or the GP list size.

^b^Population includes non-students.

### Identification

Participants described how students would differ in how they identified their candidacy for health services. Some students perceived themselves in need based on early signs they were not coping, while others only did so once unwell or even in crisis. A feature of participants’ accounts was the social context in which potential candidacy was identified. For example, students were described as identifying candidacy when comparing themselves to how they perceived other students were managing, or where they were not meeting social, cultural, and familial expectations. Other students identified candidacy after seeking explanations online, through their peers, or through other relationships:

[The student] had done lots of reading about neurodivergence and the different ways it can present and kind of self-diagnosed, and came to me with kind of a list of things that she felt were going on for her. (General Practitioner, GP1)

She wasn't managing very well to balance [different roles and tasks in the student’s life] … She was a bit like ‘OK, why can't I function if everybody else is kind of doing OK?’ … It kind of snowballed into a more of a depressive episode. (Psychologist, PSY2)

In crisis situations, identification of candidacy did happen when the student was isolated but often involved a range of other people in the student’s relational network. Accounts highlighted involvement of tutors, academics, family, or the students’ peers:

[The student] was in halls … in acute distress ... [the student] had a kind of a counsellor or I’m not quite sure what the qualification of the [university] support staff is … He [the student] had a key worker within that, but they were concerned and they’d sent him to us. (Psychiatrist, CRIS2)

Participants also described their role in identifying candidacy. This arose in situations where students’ needs could not solely be addressed in the service the student had reached. This feature of identification was strongly influenced by local operating conditions, in addition to the articulated needs of the student. For example, one psychologist spoke about the degree of complexity in the student’s presentation meant their needs could not be fully addressed in their eight-session model of care, meaning further identification occurred towards the end of treatment:

The [student] presentations are so severe that maybe by the by the end of [short term] treatment you haven't made a huge amount of headway with reducing the behaviour …. [and] make sure that the clients are kind of contained at the end of treatment so that they're aware of where else to go. (Psychologist, PSY1)

### Navigation

Students’ entry into the health system took place following identification of a need for help. Participants described students drawing on their knowledge of services they had used before. For example, university services, General Practitioners (GPs), and emergency and crisis pathway services described instances where students had received their services before. But in some cases, students used their relationships with others to assist them in finding an entry point to the health system. The role relationships played ranged from actively accompanying the student to the service to a more passive role, signposting or recommending a particular service to the student:

[The student] had sought help from a university councillor … but kind of came to me really for the letter and then we kind of had a chat [about mental health] (General Practitioner, GP3)

Friends or a tutor or someone [else] would say, ‘You [the student] need to go and see someone’. (Mental Health Nurse, CRIS3)

Awareness of a service by students was a clear feature in the data. Participants based in universities and general practice perceived their services to be well known by students and university staff. However, one GP said that university services were not always well known to students.

### Service permeability

Participants all described features of their service they thought affected service permeability for students, influencing who reached their service. Students’ concerns about confidentiality and stigma were highlighted. For example, NHS participants reported students were concerned about the consequences of the university or their family finding out they were unwell. While university participants reported students were not always willing to engage with external (i.e. NHS or other services), confidentiality concerns were not reported as a reason.

Thresholds or scope of the service shaped who reached a particular service. For example, participants working in psychological services reported situations where students presented in such a severe psychological state they were not appropriate for their service. In contrast, crisis services described how they would see anyone because their services were always open:

We have an open door. So, I guess that is very good for students. I mean, it’s 24/7 service. They can come here, we liaise quite, quite frequently with … the [university] support services (Psychiatrist, CRIS2)

Participants from university services spoke about students seeking their services, which were beyond their capability to manage. Sometimes students were ineligible for care in the NHS or lacked GP registration. These features, and the general transient nature of the student population, were described as adding additional barriers to potentially reaching NHS services:

We’re trying to get hold of [the student], trying to get her registered with the GP because if she’s not registered with the GP she can’t… access [NHS secondary care] … She’d live in one place, and she just moved out again into a hotel. (Manager, MAN1)

All participants gave accounts of international students who reached their service in crisis. These examples were particularly prominent in interviews from participants working in crisis and emergency pathway services and university services:

[The student] had written to their professor, saying that because [the professor] had failed them on an assignment that they were gonna have to kill themselves … Obviously the professor highlighted it and then [the student] got brought to our service and she'd [taken university materials] in order to complete her suicide attempt (Psychiatrist, CRIS2)

### Appearance

Participants described how students, upon reaching a health service, asserted a claim for candidacy:

[Students will] have an idea of what they want to happen often. So, it’s often about sort of meeting those expectations as well within the constraints of the NHS, which we know at the moment is hugely under stress especially GPs. (General Practitioner, GP1)

Where the student was described as unwell, greater numbers of people tended to be involved in conveying their concerns or views. For example, professionals, particularly those in crisis services, spoke about needing to draw on accounts of others because the student presentation was so severe the student could not articulate their needs, or the student was from a cultural background which meant it was difficult for professionals to have a clear idea about what was going on. Participants in university services reported a range of people (e.g. academics, professional staff, family, and friends) getting in touch with the service to express their concerns about a student.

### Adjudications

Once a student appears at a service, professionals had to make a series of judgements about the claim for candidacy. These were complex adjudications, often involving professionals seeking the expertise of the wider team within their service. Participants described a range of considerations including the claim, the student’s wishes and wider situation, knowledge about the local health system, service capacity and relationships with other services. In this sense, local operating conditions had a powerful influence on adjudication of candidacy. For example, GPs described examples where waiting lists for secondary care services played a role in their adjudication of the student’s claim:

[The student] wanted an answer for things, and I think for him the path was trying to get an ADHD and ASD [Attention Deficit Hyperactivity Disorder and Autism Spectrum Disorder] assessment. And sometimes these can be more challenging because the waiting times for both [services] are massive. (General Practitioner, GP3)

If someone needs an admission, … [this is] not about the student, it’s just about the system. It becomes really difficult because, is the halls of residence their address? Is it the parents’ address? Where should they go and then it becomes a big bureaucratic discussion. (Mental health nurse, CRIS3)

Judgements for participants working in university services were complex and had consequences for the institution. For example, one university-based psychotherapist described a student in crisis. The student was an international student who was not registered with a GP at the time, and was therefore unable to access secondary mental health services. The professional had to mobilise resources within the university to address the claim:

He [the student] wasn’t attached to a GP. So, we have this very depressed man … And we couldn’t get him to a GP because he didn’t seem to have the right to access a GP and wouldn’t access a GP. A complete nightmare … So he ended up getting everything [the university could offer]. (Psychotherapist, CPT1)

Participants said time was often crucial to making an appropriate adjudication. Mental Health Advisors spoke about meeting with a student on several occasions, each for 45min, to get to the crux of the student’s problem. Sometimes, time was lacking. GPs provided examples where they were limited by their available time.

### Offers and resistance

Participants reported that the offers of care they made to students were bounded by local operating conditions, meaning further identification of candidacy was sometimes necessary. For example, accounts from GPs described identifying further candidacy for other services unless the student’s need could be resolved. Other examples related to when the level of complexity involved with the student’s mental health problem could not be resolved within the treatment time frame. All the participants working in crisis and emergency pathway service described how their offers were made under tight time constraints, meaning it was necessary to offer further treatment in the future:

I’d say one of the interesting things about all of the students that I’ve seen has been the level of complexity. So often student services are set up to do fairly brief intervention, so short sessions of CBT [cognitive behavioural therapy] … What’s clear is that very often the treatment need is much greater than that. (Psychiatrist, PSYC2)

Students did not always accept offers of help, and the professionals’ response to this resistance varied. The contrast was most stark between accounts provided by NHS and university services. Among participants in NHS services rejections by the student meant the end of candidacy, unless the student needed to be detained under the Mental Health Act. In contrast, participants in university services said candidacy did not always end here. For example, if the professional was concerned about the student’s safety, they sometimes continued to monitor or contact the student even if the student rejected the offer for help. As one manager said about students who did not attend scheduled treatment sessions:

We’re like, … ‘What’s going on? Where is this person, you know, where is she? I’m really worried about her.’ And you have to kind of go into crisis mode and try and find them (Manager, MAN1)

### Local operating conditions

Dynamic operating conditions affected candidacy. Firstly, governance and policy influenced how NHS and universities interacted. Participants reported how these were often localised. For example, participants from one locality spoke about attempting to develop local data sharing agreement so both NHS and universities could improve coordination between two systems. Particularly among university participants, the absence and presence of policies within their organisation was perceived to influence how decisions were made, who was responsible, and level of risk the organisation held. The two university managers emphasised they were not health care providers but advice services. However, sometimes they had limited choice in meeting higher levels of demand.

We had a student who had suicidal ideation … [-which] was causing concern obviously for, you know, college staff and others - and reluctant to engage with external services … So, part of that felt like the institution is holding a level of risk there that the institution can't really hold. (Manager, MAN1)

Secondly, participants from both systems characterised coordination as piecemeal, dependent on individual professionals or necessitated in the case of a particular student:

You get an e-mail from [an academic’s office] to say, ‘Oh, we had this student in, looking to speak to the [head of department]. I wonder if she’s known to your team?’ But like, ‘Yeah, she is. Absolutely.’ She's now under every team. But just being really sure there's one person to coordinate that (Manager, MAN2)

Thresholds for entry to secondary mental health services were described by all participants as shaping candidacy. For example, GPs spoke about how students they referred could face long waiting lists or be rejected. GPs also described having to rely on university services, sometimes signposting or referring students for counselling or advice. Participants perceived these features as leading to relative selectivity in non-crisis NHS services, with ambiguity regarding who ultimately treats a student:

There’s always a difficulty in referring out because there often isn't anywhere to refer out to. You know, people have to be, like, kind of dying until they get the help … I saw [a student with a borderline personality disorder diagnosis] for probably three years. (Psychotherapist, CPT5)

## Discussion

**Figure 1.**
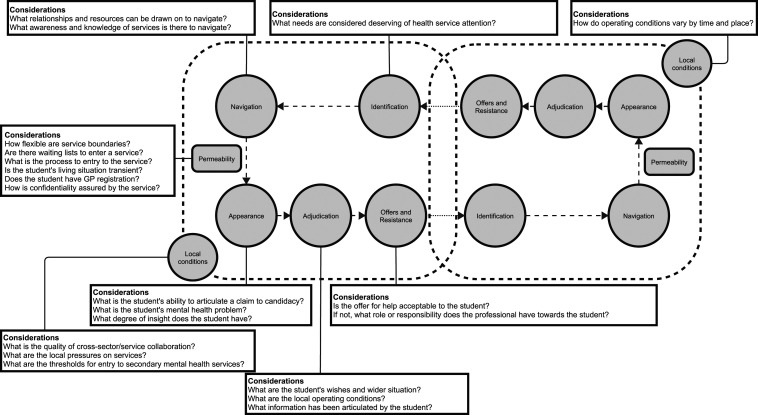
Key influences on students’ access to mental health support.

This study explored the experiences of health care professionals who interact with students experiencing mental health problems. We considered how professionals understand the presentations of students, actions they take in response, and how those actions are influenced in their work setting. The action professionals took depended on who was considered an appropriate candidate for their service, while the scope for action was shaped by the local operating conditions of the service, often described as lacking and disjointed, and what treatment offer the student considered acceptable. Our analysis suggests access to mental health support for students is highly contingent on both the dynamics of the student’s social context and the local health system. The key influences suggested by our analysis are illustrated in Figure 1.

Our analysis mirrors the wider literature around complexity of access in contemporary health systems.^2,19,26^ Participants in this study described wide-ranging student presentations, varied outcomes for students, and students reaching services through multiple pathways. Similar findings are reported in qualitative studies of academic and professionals who help students with mental health problems.^12,26^ For example, students describe differences in what needs are identified as deserving of help from a service, differences in relationships and resources students can draw upon to navigate complex care pathways, and unequal experiences reaching health services and engaging with care.^8,13–15,27^ Epidemiological literature illustrates potential consequences of these access challenges, with significant variation in use, adequacy of treatment, and treatment drop out.^9,29^

The concept of candidacy may shed light on these findings, by focusing on the intricate dynamics of identifying needs, navigating to the right support, and presenting those needs within the broader conditions that give meaning to this process. Given its origins, candidacy might also find relevance in informal settings, which are often the first point of contact for many students seeking help.^18,20,5,16^

Our analysis also highlights how the local operating conditions influence on student’s access to mental health care. This appeared to have a limiting effect of sustained collaboration between professionals. Professionals were not always aware of suitable services, limiting students’ access to appropriate support. Qualitative studies of students and other professionals also highlight the influence of local operating conditions. In particular, a lack of continuity and coordination in care.^13,14^ Fragmentation within and across services, and when transitioning between child and adult services, has been found in other studies of student mental health and across other populations.^2,13,14,28,30^ A wide body of literature has highlighted this long-term challenge in provision of integrated care.^2,19^

Further, our results suggest professionals can be uncertain about whether to treat a student as a dependent child or independent adult. Professionals described a sense of responsibility for the student, uncertainty about involving parents or caregivers in decision-making, student presentations relating to social and familial expectations, and meeting developmental challenges. The wider literature gives a context of academic competition, increasing housing/financial pressure and low staff-to-student ratios, meaning it can be increasingly difficult for some students to continue through university.^2,8^ Some epidemiological research in the US has shown associations between higher staff-student ratios and supportive environments, resulting in lower student mental distress and earlier health service utilisation.^29^

### Policy implications

Our analysis suggests simply aiming to increase service utilisation may not improve experience or outcomes for students. Candidacy highlights how gaining access to appropriate mental health support involves a range of individual stakeholders and services across a local health system. Service fragmentation and emerging adulthood may increase the complexity of student’s gaining access to appropriate mental health intervention. Policies to address fragmentation at a system level should aim to promote collaboration.^2,4,26^ This may be through developing structures and communities of practice in local geographic areas to facilitate smoother triage, referral, and sharing of expertise.^26^ Greater efforts to signpost services, information on how to access services, and self-referral mechanisms are likely to be important to reduce system level barriers.^1,8,19,20^

Students utilise multiple services during an episode of care. This suggests a role for datasets that link service use data across multiple services within a local area to inform commissioning, research and policy making.^4^ This may provide greater clarity in patterning and determinants of service use, characteristics of students who reach services, and overall adequacy of care.^26^

### Limitations

This study has four main limitations. First, our study primarily included professionals working in ‘elite’ universities across London and may have systematic population differences to other settings.

Second, the services included in this study are all free at the point of use. As such, our findings may be less relevant in settings where costs are incurred in accessing services.

Third, while our study aimed to capture a comprehensive view of a student’s help-seeking history, it might not encompass the complete narrative. Notably, students frequently turn to informal sources of support before approaching formal services.

Fourth, while candidacy provides particular insight into the role of relationships in service access, there are some limitations to this approach. It focuses on the individual and cannot consider the broader social and cultural context in which an intervention took place. We could not therefore consider economic and political factors that may influence access to mental health intervention for students. Furthermore, candidacy does not directly address power dynamics that can be involved in decision-making by professionals, such as the way medical interventions can be used to control or manipulate patient behaviour.

## Conclusions

Our study affirms the value of candidacy in understanding access to mental health services for university students. Here, access is an emergent outcome of a contingent, dynamic, and recursive process. Gaining access takes place within a context of increasingly fragmented health systems and emerging adulthood, shaping what mental health problems are viewed by students as appropriate for mental health services and what course of action professionals take. These processes may play a role in the unequal mental health outcomes observed in students. Policies and practices that aim to build and facilitate relationships between services, professionals and students, and develop services and interventions with a developmental focus, may improve outcomes for students.

## Supplemental Material


Supplemental Material - University students’ access to mental health services: A qualitative study of the experiences of health service professionals through the lens of candidacy in England
Supplemental Material for University students’ access to mental health services: A qualitative study of the experiences of health service professionals through the lens of candidacy in England by Tom G Osborn, Rosa Town, Majeed Bawendi, Emily Stapley, Rob Saunders and Peter Fonagy in Journal of Health Services Research & Policy.
