# Supplementary material for: University students’ access to mental health services: A qualitative study of the experiences of health service professionals through the lens of candidacy in England
Source: J Health Serv Res Policy. 2024 Feb 27;29(4):230–9. doi: 10.1177/13558196241235877 (PMC11346130; doi:10.1177/13558196241235877)
Supplement: Supplemental Material - University students’ access to mental health services: A qualitative study of the experiences of health service professionals through the lens of candidacy in England [file sj-pdf-1-hsr-10.1177_13558196241235877.pdf]

Journal of Health Services Research & Policy
University students' access to mental health services: a qualitative study of the experiences of health service professionals through the lens of candidacy in England
Osborn TG, Town R, Bawendi M, Stapley E, Saunders R and Fonagy P

S1: Consolidated Criteria for Reporting Qualitative Studies (COREQ)

Table 1: Consolidated criteria for reporting qualitative studies (COREQ): 32-item checklist

No. Item	Guide questions/description	Reported on Page #
Domain 1: Research team and reflexivity		
Personal Characteristics		
1. Inter viewer/facilitator	Which author/s conducted the interview or focus group?	7
2. Credentials	What were the researcher's credentials? E.g. PhD, MD	6
3. Occupation	What was their occupation at the time of the study?	6
4. Gender	Was the researcher male or female?	6
5. Experience and training	What experience or training did the researcher have?	6
Relationship with participants		
6. Relationship established	Was a relationship established prior to study commencement?	6
7. Participant knowledge of the interviewer	What did the participants know about the researcher? e.g. personal goals, reasons for doing the research	8
8. Interviewer characteristics	What characteristics were reported about the inter viewer/facilitator? e.g. Bias, assumptions, reasons and interests in the research topic	6 and 8

Domain 2: study design		
Theoretical framework		
9. Methodological orientation and Theory	What methodological orientation was stated to underpin the study? e.g. grounded theory, discourse analysis, ethnography, phenomenology, content analysis	6, 8 and 9
Participant selection		
10. Sampling	How were participants selected? e.g. purposive, convenience, consecutive, snowball	6 and 7
11. Method of approach	How were participants approached? e.g. face-to-face, telephone, mail, email	6 and 7
12. Sample size	How many participants were in the study?	11
13. Non-participation	How many people refused to participate or dropped out? Reasons?	11
Setting		
14. Setting of data collection	Where was the data collected? e.g. home, clinic, workplace	7 and 8
15. Presence of non-participants	Was anyone else present besides the participants and researchers?	7 and 8
16. Description of sample	What are the important characteristics of the sample? e.g. demographic data, date	11
Data collection		
17. Interview guide	Were questions, prompts, guides provided by the authors? Was it pilot tested?	8
18. Repeat interviews	Were repeat inter views carried out? If yes, how many?	8
19. Audio/visual recording	Did the research use audio or visual recording to collect the data?	8
20. Field notes	Were field notes made during and/or after the inter view or focus group?	8
21. Duration	What was the duration of the inter views or focus group?	7
22. Data saturation	Was data saturation discussed?	8
23. Transcripts returned	Were transcripts returned to participants for comment and/or correction?	8

Domain 3: analysis and findings		
Data analysis		
24. Number of data coders	How many data coders coded the data?	7 and 8
25. Description of the coding tree	Did authors provide a description of the coding tree?	N/A
26. Derivation of themes	Were themes identified in advance or derived from the data?	8 and 9
27. Software	What software, if applicable, was used to manage the data?	N/A
28. Participant checking	Did participants provide feedback on the findings?	9
Reporting		
29. Quotations presented	Were participant quotations presented to illustrate the themes/findings? Was each quotation identified? e.g. participant number	11-24
30. Data and findings consistent	Was there consistency between the data presented and the findings?	11-24
31. Clarity of major themes	Were major themes clearly presented in the findings?	11-24 and online supplementary materials
32. Clarity of minor themes	Is there a description of diverse cases or discussion of minor themes?	11-24 and online supplementary materials

S2: Themes derived from the data

Theme 1: Students struggling at university

Every participant recounted students who sought out their service due to daily struggles they encountered at university. This theme prominently featured two aspects: Students Trying to Fit in a University and Coping with Mounting Pressures. Participants elaborated on how these varying presentations influenced their subsequent actions.

Sub-theme 1: Students trying to fit in at university

All participants described presentations related to students' attempts to integrate into the university environment. Central to this concept was the 'interaction' between the varied backgrounds of the student body, an internally constructed yet unrealistic notion of the 'ideal student', and the prevailing university culture. This dynamic was evident in cases like a student who had achieved significantly at school, but now perceived themselves as underperforming based on an impractical expectation of their university performance. This misalignment was attributed to the influences of the new social setting and a perceived intensive academic work ethic.

> *"You [the student] might have been really good [in school] [….] you were high achiever. You've done things relatively well. You've been kind of praised. You've been seen and then you go to uni that kind of disappears and…I think that that's a big shock to a lot of people and then that often leads them to, kind of question the kind of ways that they've been living and then from that….kind of withdrawing."*

(Psychologist 1, PSY1)

Sub-theme 2: Coping with mounting pressures

All participants discussed about students who sought assistance due to an overwhelming sense of being unable to manage increasing demands in their lives. One aspect of this sub-theme was the students' perception of an escalating series of demands, manifesting in areas such as housing, finances, relationships, academics, or other challenges. Another characteristic was the often-abrupt onset the student's inability to cope with these demands, stemming from events like a relationship breakdown down, sudden financial difficulties, or other rapid shifts in their circumstances. The backdrop for these presentations often portrayed the student as socially isolated.

> *"I'd say impactful maybe on kind of her day-to-day. And because then things weren't getting better, because when she was kind of low that first time, she kind of slowed down all the things that she was doing, her workload kind of piled up. She felt kind of more distant from friends. And then she was spending more time at work and not really doing anything else."*

(Psychologist, PSY2)

> *"He was an international student, so he was not Chinese actually […..] he felt a lot of pressure from home […..]. Basically, it was a breakup. It's a girl […] he'd started to fixate on, I think, and he wasn't psychotic. It wasn't a psychotic disorder. I think it was, it was a young man who sort of got a bit out of his depth and was outside of everything he understood."*

(Psychiatrist, CRIS2)

Theme 2: Students with a diagnosable mental health problem

Consistent with all the conceptualised themes, each participant mentioned student presentations which they attributed to an identifiable mental health condition. Two salient aspects of this theme were the Complexity of the Problem and the Severity of the Presentation.

Sub-theme 1: Complexity of the Problem

This sub-theme elucidated the different ways all participants perceived the mental health challenges students presented with as multifaceted. A prevailing notion related to the student's mental health history, highlighted by descriptions of students who disclosed multiple diagnoses, medication regimes, or significant traumatic histories. The notion of complexity was further underscored by behaviours the student exhibited, such substance abuse, self-harm or engaging in 'chemsex'. Their social contexts adder another layer, with participants noting intricate or distressing social relationships linked to the students' predicaments. Cultural facets also contributed, exemplified by a professional recounting a student's psychotic episode, which proved challenging to fully understand and address due to cultural nuances. Often, participants contextualised this complexity against the backdrop of the student's developmental stage. The multifaceted nature was seen both as a hallmark of the mental health issues students presented to the professional and as a factor influencing the feasibility of intervention strategies.

> *"[There is often] a massive overlap between something like that [personality disorders] and complex PTSD and [those] kind of needs…Uh, disentangling, you know detangling [what's going on]. And because they can present us, you know, having quite severe personality issues, umm difficulties. But it could be just kind of complex PTSD."*
>
> (Manager, MAN2)

> *"And [she] had been [seriously sexually assaulted when she was a teenager] and that, of course, had an impact on her being able to form personal relationships, so she, you know, found it very hard to, to trust people. [….] She had features of clinical depression. She had disturbed sleep, and she had a low mood with a what's called a diagonal variation. So, you know, that mood changed over the day [….] she was socially isolating, and she was very fearful."*
>
> (Psychiatrist, PSYC1)

Sub-theme 2: Severity of the Presentation

Every participant highlighted the element of severity when recounting student mental health presentations. These ranged from milder presentations to those people who were acutely psychotic and suicidal. Some professionals described milder presentations as early symptoms or manifestations of more prevalent mental disorders, linked with recent yet notable dysfunction in daily life. In contrast, the more intense presentations were characterised by acute suicidal tendencies, psychosis or mental health challenges that necessitated hospital admission or detention under the

Mental Health Act. The severity was a determinant both in understanding the student's mental health issues and in deciding an appropriate intervention.

> *"The person [was] becoming quite the fragmented, and so the person was in the early stages of psychosis and so like moving away from home, really struggling with living, becoming disorganized and things are falling apart and we [were] seeing that sort of thing."*
>
> (Mental Health Nurse, CRIS3)

Theme 3: Collaborative Capacity-Building

The predominant theme derived from the data was Collaborative Capability-Building. Every professional recounted strategies they adopted both to assist the student and to facilitate their own professional intervention, all aimed at enabling the student to progress in life. Two principal facets of this theme were The Student-Professional Relationship and Professional Collaboration with Other Stakeholders for Student Support.

Sub-theme 1: The Student-Professional Relationship

Each account underscored the mutual relationship between the professional and the student, expressed variably across the dataset, typically predicated upon the professional's expertise and the student's unique challenges. A salient aspect was the need to appreciate the student's circumstances to determine apt solutions or interventions and to afford the student the necessary space and understanding. Professionals relied on diverse sources of information, including direct communication with the student, supplementary assessment data, and their

relational approach, which was often described as curious, responsive, trust-building, while establishing clear expectations.

> *"[I] listen first of all, to let that person know that they're feel that that someone has taken their concerns on board and taking them seriously and that. And that, makes some, some little plan agreed [….] I'll see the student again"*
>
> (Mental Health Advisor, MHA1)

The intricacy of the student's situation, their level of self-awareness, and expectations were deemed pertinent in guiding the professional's response. Another facet of this sub-theme centred on the spectrum of interventions as delineated by the professionals. Their actions were anchored in their specific role, obligations, expertise, and past experiences. For instance, a psychologist discussed offering structured therapeutic interventions, while a mental health advisor focused on decomposing challenges into manageable segments, subsequently strategizing with the student on practical solutions. Universally, professionals emphasised the indispensable role of student participation, acknowledging that solutions required joint effort.

> *"So often the treatment is, is about building up confidence to be able to manage the situations that the addictive behaviour is being used to manage so helping to build our self-esteem helping the person to reflect on what they do bring to us [the student] a social situation that's valuable helping them."*
>
> (Psychologist, PSY2)

Sub-theme 2: Professional Collaboration with Other Stakeholders for Student Support.

Every participant highlighted the inclusion of external parties in their interventions. This approach manifested differently throughout the dataset. One distinct element was the degree to which these external individuals or entities were engaged, from proactive to more reactive measures. For instance, the incorporation of family members, friends, or peer networks in supporting the student was often viewed as a more reactive approach.

> *"So I said, 'well, I think your dad would rather know that you're actually talking to a psychiatrist and acutely distressed and even possibly suicidal. And that your dad would rather know about that. And I'm sure he could afford to stump up for the plane ticket in that situation'. But there was just no way this he [the student] was like, 'well, that's not a possibility. So, I'm not going to be asking him'".*
>
> (Psychiatrist, CRIS2)

On the other hand, involving fellow professionals spanned from immediate contact with other services in response to a student's particular issue to pre-arranged discussions or soliciting the expertise of others – such as in supervisory sessions or multidisciplinary team meetings. Another aspect of this sub-theme was the objective behind these collaborations. They were initiated either as interventions to aid students or professionals by drawing on the support of individuals like family, friends, or specialists via discussions or referrals. Such collective efforts were also crucial for

understanding the student's circumstances more holistically or for determining a more effective intervention strategy.

> *"We are very fortunate. We also have a psychiatrist who worked with us. Would that gives us a very and we can ring her. [….] You have other support as well, and of course [in the university] is we are near the A&E, you can always take them straight to A&E we wanted to while he was concerned."*
>
> (Psychotherapist, CPT3)

Theme 4: Relational Network Resources

All participants conveyed that their chosen actions were influenced by the available resources within the collaborative network supporting the student. One primary aspect was the quality and nature of relationships involved.

> *"You know, and, and all, all of us have individual supervisors are in in group supervision so, so and that we all use external supervisors so that it doesn't get caught up kind of in any kind of team, team dynamics."*
>
> (Psychotherapist, CPT3)

Another dimension was the variety of resources accessible to the student and the professional. This encompassed areas such as expertise, understanding of the current student experience and local health system, time, emotional capacity, influence wielded by the professionals, as well as financial and tangible assets. Descriptions using terms like localised, insufficient, limited, fleeting, reactive, and fragmented frequently appeared. These elements collectively dictated the feasible

outcomes in a situation. For instance, a GP remarked on the constrained time during consultations, necessitating leveraging the resources of other services or the student's personal networks due to existing rapport.

> *"So we're constantly triaging and assessing. And deciding what's appropriate to go to secondary care, and particularly in mental health, where the resource within the mental health services are so low that so much of what we even refer gets rejected, let alone all the other stuff we don't refer. You're constantly thinking, you know, 'is, is this patient even gonna get to the point where they're gonna see someone and they gonna meet the criteria?'".*
>
> (General Practitioner, GP1)

Theme 5: Systemic Health Structures

Participants universally indicated that the systemic features of the health system swayed the course of action. These influences manifested diversely. One primary area was the organisational structures or pathways bridging universities and the NHS. Such structures often came across as disjointed or insufficient. This was evident, for example, in the limited communication and data sharing protocols between the NHS and academic institutions, even though these are vital for efficient care. The decision-making processes at the service and organisational levels emerged as another critical aspect, determining the clarity and consistency of actionable plans.

> *"You know, multiple responsibilities that aren't necessarily spelt out, so it kind of almost lands a little bit in your inbox and you go 'oh, OK, So what I'm going*

> *to do here?' OK. So then landing on my feet or a little bit and kind of going. OK, So what do I know? What don't I know? How can I solve this?"*
>
> (Psychotherapist, CPT5)

Subsequent elements concerned the service structure in the NHS, spanning areas like access, evaluation, and continuity. These elements impacted both the opportunities students had to engage with professionals and the range of interventions available to the latter. Referral structures, often specific to geographic regions and distinct health systems, influenced the ease and nature of services students could access. Some professionals underscored challenges with referrals due to students' mobility or stringent criteria. Linked with this was the funding paradigm and the necessity of GP registration for students. Collectively, the portrayal was of a reactive, disjointed system. This often resulted in university professionals bearing considerable risk, whilst NHS staff remained uncertain about the resources available within academic institutions.

> *"If someone needs an admission […] that's not about the student, that's just about the system. It becomes really difficult because is the halls of residence their address? Is it the parents address? Where should they go? And then it becomes a big bureaucratic discussion that's hard but….that's not about the person."*
>
> (Mental Health Nurse, CRIS3)

S3: Features of Candidacy

Candidacy is defined as:

> "The ways in which people's eligibility for medical attention and intervention is jointly negotiated between individuals and health services. ... [It] is a dynamic and contingent process, constantly being defined and redefined through interactions between individuals and professionals, including how 'cases' are constructed. Accomplishing access to healthcare requires considerable work on the part of users, and the amount, difficulty, and complexity of that work may operate as barriers to receipt of care. The social patterning of perceptions of health and health services, and a lack of alignment between the priorities and competencies of disadvantaged people and the organization of health services, conspire to create vulnerabilities".[18]

Table 2: Liberti et al's definition of Candidacy for Mental Health Services[20]

Construct	Description
Identification of candidacy	How people make judgements about seeking help for their mental health from services (which need not be exclusively medical) is important to whether they see themselves as candidates who need, deserve, and are entitled to attention and care. These judgements can be strongly impacted by macro-level changes that mean people internalise views of what constitute "worthy" objects of care and attention.
Navigation of services	Using services demands that service users and their carers have knowledge of services and how they operate, the social capital necessary to navigate the system, and access to the material and practical resources needed to find a point of entry to services.
Permeability of services	The ease with which service users and their carers can use mental healthcare services depends on how well configured services are to meet the specific needs of those with mental health difficulties, the criteria being used, the degree of cultural alignment, and number and type of barriers to access.
Appearance at services	Appearing at services involves people making a claim to candidacy. It demands a set of competencies and socio-cultural alignments that may be particularly challenging for those with mental health difficulties.
Adjudications by professionals	Professional judgements about candidacy strongly influence access to attention and interventions, and depend in part on a repertoire of judgements. Operating conditions and resource constraints may powerfully constrain these judgements and may at times result in moral injury.
Offers and resistance	Offers of care may be made that may be accepted or

	refused by individuals, sometimes because the care offered is not seen as helpful or acceptable. Recursivity – previous experiences of poor care – may be an important influence.
Operating conditions and the local production of candidacy	Macro-structural influences have major impacts on people's candidacy for care, and reconfigurations of systems may be particularly consequential not only in restricting availability of supply, but also in refiguring how individual service users, carers and staff construct people's eligibility care.
